# The performance of plant essential oils against lactic acid bacteria and adverse microorganisms in silage production

**DOI:** 10.3389/fpls.2023.1285722

**Published:** 2023-11-10

**Authors:** Lijuan Chen, Xi Li, Yili Wang, Zelin Guo, Guoming Wang, Yunhua Zhang

**Affiliations:** ^1^ College of Animal Science and Technology, Anhui Agricultural University, Hefei, China; ^2^ College of Resources and Environment, Anhui Agricultural University, Hefei, China

**Keywords:** essential oil, active ingredient, bacteriostatic, fungistatic, silage

## Abstract

Plant essential oils have played an important role in the field of antibiotic alternatives because of their efficient bacteriostatic and fungistatic activity. As plant essential oils are widely used, their activity to improve the quality of plant silage has also been explored. This review expounds on the active ingredients of essential oils, their bacteriostatic and fungistatic activity, and mechanisms, as well as discusses the application of plant essential oils in plant silage fermentation, to provide a reference for the development and application of plant essential oils as silage additives in plant silage fermentation feed.

## 1 Introduction

Considerable attention has been paid to the application of plant extracts in livestock and poultry production as alternatives to banned additives such as antibiotics. Plant extracts is a mixture of natural compounds or components extracted from plant materials. Due to the presence of numerous bioactive compounds with pharmacological properties, they have great potential for research. Moreover, they are considered a sustainable and eco-friendly choice due to their natural, biodegradable nature, and their ability to reduce reliance on synthetic chemicals. Huge scientific studies regarding the application of plant extracts in silage preservation have reported the potential antifungal agents from this enriched flora (Cock and Van Vuuren, 2015), Aloe vera extract has a wide range of microbial growth inhibition activities, and it has been reported to have a significant inhibitory effect on the mycelial growth and spore germination of *Penicillium italiana* (Zapata et al., 2013). The organic easy extract of tea plant contains a variety of natural non-ionic surfactants, which can cooperate with some antibacterial agents to antagonize fungi (Hao et al., 2010). Some studies have reported that the ethanol extract of *Ficus hirta Vahl* exhibits a fungistatic effect against *Penicillium tilikum* (Wan et al., 2017). As an important class of plant extracts, an increasing number of studies have shown that plant essential oils have significant antibacterial activity, which makes them more attractive to researchers. Commonly used plant essential oils include thyme essential oil, clove essential oil, cinnamon essential oil, oregano essential oil, mint essential oil, and curcumin essential oil. At present, plant essential oils are primarily used to maintain animal health, improve animal performance, and enhance the quality of livestock products in the breeding industry. With the deepening of research on essential oils, the function of plant essential oils in improving the fermentation quality of feed silage has been explored (Matté et al., 2023).

## 2 Main active component of plant essential oils

Plant essential oil is a chemical substance extracted from the bark, peel, leaves, buds, seeds, flowers, and other parts of plants by steam distillation, solvent-assisted extraction, hydrogenation distillation, ultrasonic-assisted extraction, supercritical fluid extraction, and solvent-free microwave extraction (Kant and Kumar, 2022). The active components of plant essential oil are divided into four categories in accordance with their structure: terpene compounds, aromatic compounds, aliphatic compounds, and sulfur-containing and nitrogen-containing compounds (Shao et al., 2020).

**Figure 1:**
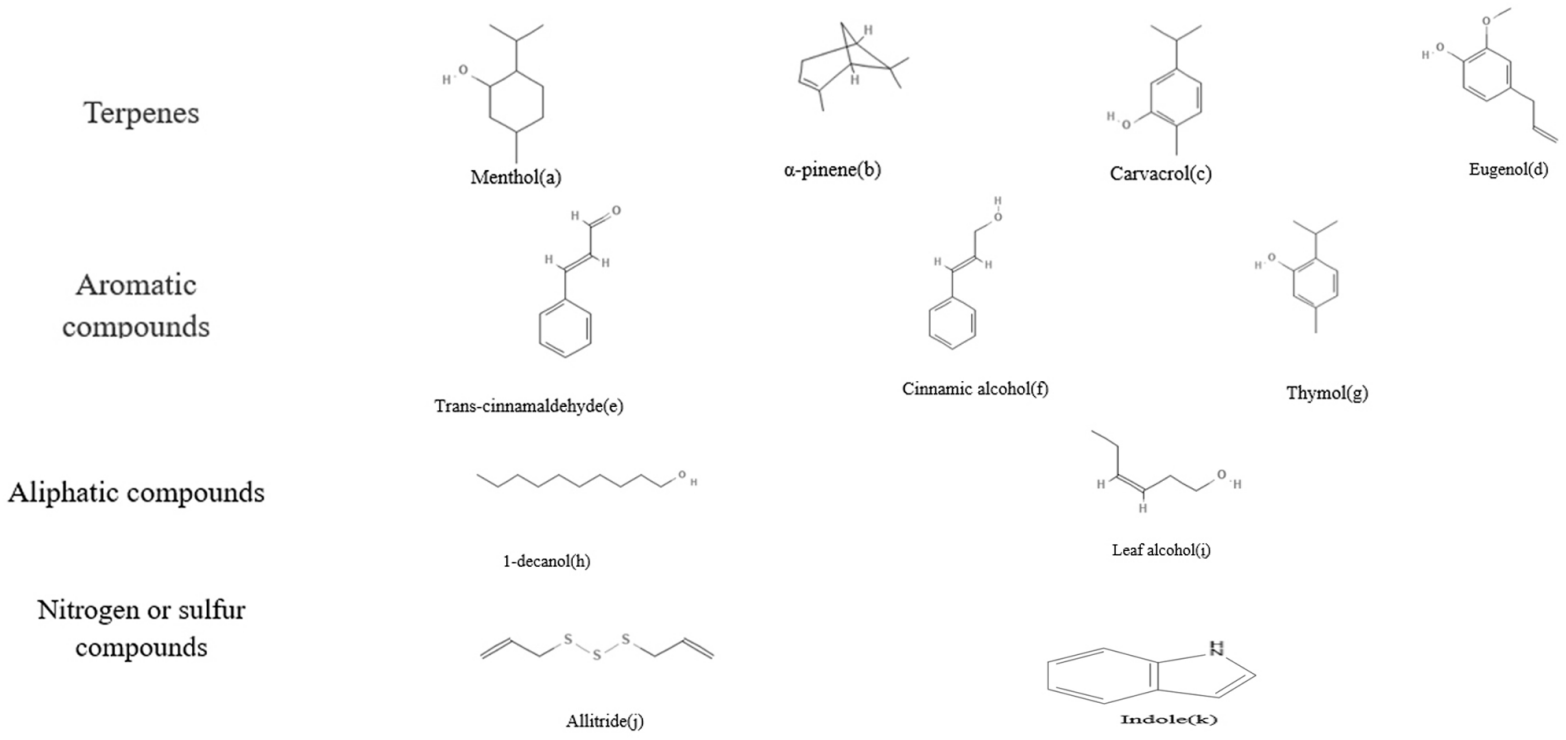
Chemical structure formula of plant essential oils.

Terpene compounds are the most common chemical components in essential oils. They are generally chain or cyclic olefins with the general formula (C_5_H_8_)_n_ **Figure 1; PubChem**, such as menthol (a), menthone, neomenthol, and isomenthone in peppermint essential oil (Zhao et al., 2022); α-terpinene (b) and myrcene in rosin essential oil (Couladis et al., 2003); carvacrol (c) and eugenol (d) in oregano and clove essential oil (Milovanović et al., 2009); and eucalyptol in rosemary essential oil (Jiang et al., 2011). The second common chemical component is aromatic compounds, which are a class of compounds with a benzene ring structure related to the fragrance of essential oils, such as cinnamaldehyde (e) and cinnamic alcohol (f) in cinnamon essential oil (Vasconcelos et al., 2018), as well as thymol (g) in thyme essential oil (Kim et al., 2022). Aliphatic compounds, which have the smallest relative molecular mass but are widely present in plant essential oils, are organic compounds composed of hydrocarbon chains or their derivatives, such as n-decyl alcohol (h) and leaf alcohol (i). Sulfur and nitrogen-containing compounds, such as allitride (j) and indole (k), are present in small amounts, but t hese compounds have extremely strong odors and characteristic aromas (Saad et al., 2013).

## 3 Bacteriostatic and fungistatic activity and mechanism of plant essential oils

The bacteriostatic and fungistatic effect of common plant essential oils such as thyme, clove, cinnamon, oregano, peppermint, and curcumin is related to their active ingredients. Each active ingredient exerts its bacteriostatic and fungistatic effect through its functional groups independently or synergistically. Therefore, the bacteriostatic and fungistatic mechanisms of different types of essential oils are distinct (Evangelista et al., 2022).

### 3.1 Bacteriostatic and fungistatic activity of common essential oils

#### 3.1.1 Main bacteriostatic and fungistatic components and activities of essential oils

​The common bacteriostatic and fungistatic active ingredient in thyme essential oil is thymol, chemically known as 5-methyl-2-isopropylphenol, which is a natural monoterpene. Thymol has the strongest inhibitory effect against molds, and 0.01–0.05 mg/mL of thymol is found to be effective against molds such as *Aspergillus niger*, *Aspergillus flavus*, and *Aspergillus fumigatus*. In addition, thymol can effectively inhibit Gram-positive pathogens such as *Bacillus subtilis* and *Staphylococcus aureus* between 0.06 and 0.2 mg/mL, while the inhibitory concentration for lactic acid bacteria must reach 0.1–0.5 mg/mL (Marchese et al., 2016). Another highly effective bacteriostatic and fungistatic active substance in thyme essential oil is terpinene, which can exhibit an bacteriostatic and fungistatic effect equivalent to 70% of streptomycin at a concentration of 0.07 mg/mL against *Phytophthora capsici* (Yang et al., 2022). Thyme essential oil (*Thymus kotschyanus*), as a mixture of multiple bacteriostatic and fungistatic active substances, has a minimum inhibitory concentration of 0.0625 mg/mL against common molds such as *A. niger*, *A. flavus*, and *Fusarium oxysporum* in silage feed or raw materials (Ownagh et al., 2010), and the minimum inhibitory concentration for yeast is 0.5 mg/mL. The inhibitory concentration for some lactic acid bacteria such as *Lactobacillus brevis*, *Lactobacillus plantarum*, and *Lactococcus lactis* must reach at least 3–10 mg/mL (*Thymus vulgaris ct.linalol*, *Thymus serrulatus* and *Thymus schimperi Ronniger*) (Gutierrez et al., 2008; Damtie and Mekonnen, 2020; de Oliveira Carvalho et al., 2020). Thus, whether the active ingredient is single or a mixture in thyme essential oil, it shows low inhibitory concentration against pathogens and high inhibitory concentration against lactic acid bacteria. This selective inhibition laid the foundation for its use in silage feed (Damtie and Mekonnen, 2020).

The main active ingredient in clove essential oil is eugenol, chemically known as 4-allyl-2-methoxyphenol, which belongs to the terpene class of compounds and has a broad-spectrum antibacterial activity against Gram-negative and Gram-positive bacteria (Khalil et al., 2017). The minimum inhibitory concentration of eugenol for Gram-positive bacteria such as *S. mutans* and *S. aureus* is generally between 0.1 and 0.2 mg/mL. Its minimal inhibitory concentrations against Fusarium species commonly found in silage, such as *Fusarium avenaceum*, *Fusarium graminearum* and *Aspergillus nidulans*, ranged from 0.1 mg/mL to 0.14 mg/mL. The minimum inhibitory concentration against *Saccharomyces cerevisiae* is 0.25 mg/mL, whereas the minimum inhibitory concentration for lactic acid bacteria such as *Lactobacillus casei* must reach 1 mg/mL (Marchese et al., 2017). Low levels of eugenol (below the minimum inhibitory concentration) show strong inhibitory activity against the expression of the physiological functions of microorganisms and the production of toxins such as ochratoxin (Jiang et al., 2022). Clove essential oil also exhibits a remarkable bacteriostatic and fungistatic activity, with a minimum inhibitory concentration of 0.2–0.3 mg/mL for yeast and a minimum inhibitory concentration of 0.031, 0.031, and 0.25 mg/mL for fungi such as *F. graminearum*, *Rhizopus stolonifer*, and *Penicillium crustosum*, respectively, which is lower than its minimum inhibitory concentration for lactic acid bacteria such as *Lactobacillus fermentum* and *Lactobacillus lactis* (*Syzygium aromaticum (L.) Merr. and L. M. Perry*) (Sameza et al., 2016; Sharma et al., 2017; Chan et al., 2018). The low inhibitory concentration of clove essential oil against harmful microorganisms and high inhibitory concentration against lactic acid bacteria determines its use in later production.

The bacteriostatic and fungistatic activity of cinnamon essential oil is derived from cinnamaldehyde, which has the strongest bacteriostatic and fungistatic activity among other secondary metabolites. Cinnamaldehyde has shown antifungal activity against *Candida* (Choonharuangdej et al., 2021), *A. flavus* (Achar et al., 2020), *F. graminearum* (Gwiazdowska et al., 2022), *Aspergillus ochraceus* (Wang et al., 2018), and other fungi, as well as broad-spectrum antifungal activity against *Escherichia coli* and *S. aureus* (Zhang et al., 2016). Compared with other active components of plant essential oil, cinnamaldehyde has the strongest inhibitory activity on the synthesis of aflatoxin and ochratoxin. The minimal inhibitory concentration of cinnamaldehyde against *F. oxysporum* and *F. gramineum* is 0.8 mg/mL, and its minimal inhibitory concentration against spoilage yeast is 0.31–1.25 mg/mL (Muñoz‐González et al., 2022). However, its minimal inhibitory concentration against lactic acid bacteria varies, with a minimal inhibitory concentration of 5 mg/mL against *Lactobacillus sakei* and a minimum inhibitory concentration of 50 mg/mL against *Lactobacillus brucei* (Sameza et al., 2016; Sharma et al., 2017; Muñoz-González et al., 2022). Cinnamon essential oil also shows good application effects. Cinnamon essential oil inhibits *E. coli*, *S. aureus*, and *Listeria* at a concentration of 0.3–0.5 mg/mL (*Cinnamomum zeylanicum*) (Nematollahi et al., 2020), and its minimum inhibitory concentration against *Penicillium citrinosus* and *Penicillium expandatum* is between 0.4 and 0.5 mg/mL (*Cinnamomum cassia*) (Lucas-Gonzalez et al., 2023). However, the minimum inhibitory concentration against lactic acid bacteria is approximately 10 mg/mL (*C. zeylanicum*) (de Oliveira Carvalho et al., 2020).

​The main active ingredients in oregano essential oil are carvacrol and thymol. Thymol has been elaborated in the previous section, and carvacrol is also a class of phenolic compounds with strong bacteriostatic and fungistatic activity. The minimum inhibitory concentration of carvacrol against *S. aureus* is 0.3 mg/mL; the minimum inhibitory concentration of carvacrol against *Streptococcus* is 2.5 mg/mL, and its minimum inhibitory concentration against Gram-negative bacteria such as *E. coli* and *Salmonella typhimurium* is less than 0.25 mg/mL (Marinelli et al., 2018; Rathod et al., 2021). The minimum inhibitory concentration against a variety of yeasts is approximately 0.2 mg/mL. On the contrary, oregano essential oil containing a mixture of carvacrol and thymol shows considerable synergistic bacteriostatic and fungistatic effects. Its minimum inhibitory concentration against a variety of *Fusarium species* is less than 0.8 mg/mL, and its antifungal activity is mostly between 0.2 and 0.3 mg/mL, whereas its minimum inhibitory concentration against a variety of lactic acid bacteria is between 5.5 and 13 mg/mL (*Origanum vulgare L.*) (Gutierrez et al., 2008; Konuk and Ergüden, 2017; Sharma et al., 2017). The bacteriostatic and fungistatic activity of oregano essential oil against lactic acid bacteria is lower than that against pathogenic fungi. Furthermore, oregano essential oil can inhibit the biosynthesis of aflatoxin after it acts on aflatoxin (*Origanum majorana L.*) (Chaudhari et al., 2020).

Peppermint essential oil has been used as a preservative in the food industry for a long time, and its bioactive substances have good effects on inhibiting pathogenic bacteria and spoilage microorganisms, such as menthol, menthone, and neomenthol (Kazem et al., 2011). A number of reports have shown that peppermint essential oil inhibits the cell activity of *S. aureus* (Kang et al., 2019), *Candida albicans*, *Pseudomonas aeruginosa* (Mahboubi and Kazempour, 2014), *Helicobacter pylori*, and *Salmonella enteritidis* (Imai et al., 2001). The *in vitro* anti-aflatoxin concentration of menthol is 1.0 mg/mL, while menthol stereoisomers and menthone do not show evident antitoxin function, which is related to their structural types (Ownagh et al., 2010; Muñoz-González et al., 2022). Peppermint essential oil has weaker bacteriostatic and fungistatic activity than menthol. Peppermint essential oil has a minimum inhibitory concentration of 62 mg/mL against a variety of mold species and more than 150 mg/mL against lactic acid bacteria, but it has considerable bacteriostatic and fungistatic activity against *S. cerevisiae*, with a minimum inhibitory concentration of 1 mg/mL. The results show that antifungal sensitivity to mold and yeast is higher than sensitivity to lactic acid bacteria. (*Mentha Piperita*) (de Oliveira Carvalho et al., 2020).

Turmerone is a glycosides active substance derived from turmeric essential oil. It mainly includes ar-turmerone and β-turmerone. Given its special structure, it has strong antibacterial and antifungal activity. At a concentration of 0.1% ar-turmerone, this purified compound reduced the growth of *Fusarium semitectum*, *Aspergillus ochraceous*, and *Calletotrichum musae* by 70%, 55%, and 68%, respectively (Dhingra et al., 2007). At 1 mg/disk, ar-turmerone strongly inhibited the growthl of C perfringens and moderately inhibited the growth of *E. coli* without any adverse effects on the growth of four lactic acid bacteria(Lee, 2006). Given the synergistic effect of various bacteriostatic and fungistatic active substances in turmeric essential oil, the bacteriostatic and fungistatic activity of the mixture is remarkably enhanced. The minimum inhibitory concentration of turmeric essential oil to various yeasts is between 0.01 and 0.03 mg/mL (*Curcuma longa L.*) (Konuk and Ergüden, 2017), and the concentration of turmeric essential oil has a remarkable inhibitory effect on Fusarium verticillium at 0.072 mg/mL (*C. longa*) (Avanço et al., 2017). However, the minimum inhibitory concentration against Lactobacillus is as high as 11–14.5 mg/mL (*C. longa*) (Khorsandi et al., 2018).

All of the above mentioned essential oils show strong inhibition against yeast, fusarium, and other pathogenic bacteria but relatively weak inhibition against lactic acid bacteria. This unique bacteriostatic and fungistatic property of essential oil contributes to the inhibition of spoilage bacteria in silage and to the improvement of silage quality.

#### 3.1.2 Synergistic bacteriostatic and fungistatic activity of essential oils

The synergistic bacteriostatic and fungistatic effects of plant oils have been gradually recognized with the exploration of their bacteriostatic and fungistatic properties. The bacteriostatic and fungistatic activity of plant essential oils results from interactions among different components, often by terpenoid compounds with the strongest internal bacteriostatic and fungistatic properties, as well as by a variety of compounds of different classes,For instance, there are interactions between compounds such as citral and thujone, camphor, ethyl acetate of borneol, and citronellal. Similarly, interactions between α-pinene and thujone, camphor, citral, citronellal, and geraniol occur. Moreover, the interaction of cineole oxide with compounds like thujone, hexanal, and hinokitiol also demonstrates a synergistic enhancement of cytotoxicity(Wright et al., 2007), and the fractional inhibitory concentration of each component against bacteria is markedly reduced. The mixed use of eugenol and linalool in basil essential oil shows higher bacteriostatic, fungistatic and antioxidant activity to some fungi and bacteria than each component itself (Juliani et al., 2009). The bacteriostatic and fungistatic active components of cinnamon essential oil, cinnamaldehyde and cinnamic acid, also show bacteriostatic and fungistatic properties when combined. The combination of components derived from different essential oils also shows synergistic effects, such as the chimerism of cinnamaldehyde and carotenoids to form a mixture of solid and liquid fats to cause defects in the structure of the lipid matrix and improve the bioavailability and retention ability of active ingredients (Procopio et al., 2022). The combination of cinnamon essential oil and clove essential oil also has synergistic effects, and the enhanced essential oil remarkably inhibits biofilm formation, destroying the cell wall structure and scavenging free radicals (Yen and Chang, 2008). The same study found that the combination of thyme and rosemary as well as the triple combination of thyme, rosemary, and cinnamon show synergistic effects on *Bacillus cinesulata* and *Pseudomonas ectrosp* (Nikkhah et al., 2017). The combination of oregano and thyme essential oils, bay and almond essential oils, and basil and thyme essential oils all showed synergistic bacteriostatic and fungistatic effects, with minimum inhibitory concentrations reduced by up to 128 times.

### 3.2 Bacteriostatic and fungistatic mechanism of plant essential oils

Most of the current studies have shown that the main bacteriostatic and fungistatic mechanism of plant essential oils is achieved by destroying the integrity of biofilm, destroying and inhibiting cell wall biosynthesis, damaging membrane proteins, and inhibiting mitochondrial function.

#### 3.2.1 Destruction of biofilm integrity

​The bacteriostatic and fungistatic effects of essential oils are directly related to their lipophilicity. The six-carbon aromatic phenol group of cinnamaldehyde allows it to cross the phospholipid bilayer of the bacterial cell wall and bind to the inner and outer membrane proteins, thereby preventing them from performing their normal function. Altered membrane permeability and loss of functional proteins that transport molecules and ions perturb microbial cells, which leads to cytoplasmic coagulation, enzymatic denaturation, and loss of proteins, as well as loss of metabolites and ions (Suxia Shen et al., 2015). Cell membrane damage by essential oil is usually manifested as the change in cell membrane surface structure (usually wrinkled or irregular), the increase of relative electrical conductivity, the decrease of membrane potential, the increase of extracellular nucleic acid and protein concentration, the change of membrane potential and conductivity, and the accumulation of intracellular active components of essential oil, which leads to the acidification of the cell membrane and the damage of the cell membrane caused by protein denaturation (Trinh et al., 2015).

Cellular ion leakage is also an important bacteriostatic and fungistatic mechanism of plant essential oils. The α- and β-unsaturated bonds of various cinnamaldehydes can be conjugated to the plasma membrane calcium ATPase on the fungal plasma membrane, which opens the ion pathway, induces Ca^2+^ efflux, and inhibits fungal activity (Hu et al., 2013). Under ergosterol inhibition, celery essential oil acts on the cell membrane of *A. flavus* to take part in the α-demethylation of lanosterol, and Ca^2+^ leakage is observed. Mg^2+^ influx is enhanced, leading to the depletion of nutrient uptake, inhibition of nucleic acid synthesis, and inhibition of ATPase-dependent respiratory activity, thereby leading to cell lysis (Das et al., 2019). *P. capsici Leonian* and *A. flavus*, which have no ergosterol production ability, and ethylene glycol bis (2-aminoethyl ether) tetraacetic acid elution treatment were used to exclude the influence of exogenous Ca^2+^. The cells showed Ca^2+^ outflow and ergosterol reduction after treatment with cinnamon essential oil, fenestra essential, oil and peppermint essential oil (Dwivedy et al., 2017). Ca^2+^ is the most prevalent regulator in the whole living system, which is involved in the regulation of the proliferation, differentiation, and apoptosis of organisms. In fungi, Ca^2+^ regulates spore formation, spore germination, hyphal branching, apical growth, and structural differentiation (Shreaz et al., 2016). The disorder of Ca^2+^ may interfere with the amount of circulating calcium ions in the mitochondria and activate the mitochondrial permeability transition pore, thereby leading to the initiation of the fungal apoptosis program (Berridge et al., 2000).

#### 3.2.2 Reduction of quorum sensing

Quorum sensing (QS) is a feedback intercellular communication system of bacteria based on the secretion and sensing of external signaling molecules. The formation of biofilms is highly correlated with density-dependent QS propagation, which affects the production of bacterial secondary metabolites and regulates the secretion of virulence factors. Bacteria in the form of biofilms are different from planktic bacteria because their association with abiotic surfaces generates a three-dimensional organizational structure that is protected from the threat of being killed by fungicides, disinfectants, and antibiotics. Bacterial cells produce extracellular polymeric substances, proteins, and extracellular DNA, which support structural stability and improve substrate exchange and nutrient cycling in biofilms (Tan et al., 2019). The inhibition of intercellular communication between bacteria and biofilm formation is considered as an bacteriostatic and fungistatic pathway of plant essential oils (Bo et al., 2023). After applying cinnamaldehyde to the biofilm, the hydrophobicity of the cell membrane decreases, resulting in the reduced adhesion of the hydrophobic surface to the biofilm surface. Aggregation also decreased, whereas self-aggregation formed the complete biological structure of several different biofilm strains, which played an important role in biofilm stability (Yu et al., 2020). Molecular docking analysis showed that cinnamaldehyde downregulated the expression of cellulose synthase (BcsA) and transcription activator protein (luxR) receptor genes, thereby inhibiting the synthesis of signaling molecules in QS (Liu et al., 2021). Diallyl disulfide in garlic essential oil inhibits the virulence factors of *P. aeruginosa* at MIC concentrations by affecting the transcription of key genes in three different QS systems (Li W.-R. et al., 2018). Carvacrol binds to homoserine lactone synthase (Expl) and transcriptional regulators (Khan et al., 2017), which in turn inhibits the production of QS signaling molecules and the expression of QS-controlled genes (Joshi et al., 2016). Eugenol inhibits protease, pyocyanin, pyranan biosynthesis, extracellular polysaccharide, and rhamnolipid and closely binds to the synthesis of carbonyl N-coa acylation regulatory protein (LasR) by *P. aeruginosa*, thereby leading to the inhibition of QS (Rathinam et al., 2017). Cyclic diguanosine monophosphate (C-di-GMP) is considered as a key cytoplasmic signal and second messenger that controls bacterial virulence, cell cycle reproduction, motility, and other behaviors, such as the biofilm life cycle in several bacteria. Cinnamaldehyde carbon can affect the level of nitric oxide by regulating C-di-GMP, thereby inducing biofilm dispersion (Topa et al., 2018), whereas the ginger essential oil test found that cinnamaldehyde carbon promoted the degradation of proteins with EAL (*Glu-Ala-Leu*) or HD-GUP (*His-Asp-Gly-Tyr-Pro*) domains. This domain is directly associated with C-di-GMP levels, thereby inhibiting biofilm formation (Kim and Park, 2013).

#### 3.2.3 Inhibition of cell wall formation

​The cell wall is an important structure in fungi, indispensable for maintaining shape integrity and fluidity, for interacting with its surroundings, and for regulating the fungal membrane. Chitin, as a scaffold for the cell wall, imparts integrity and strength to the cell wall, thereby offers protection against external stresses, mechanical damage, and immune responses from hosts (Baker et al., 2007). Glucans attached to chitin serve as attachment points for other structures, constituting amorphous outer and intermediate fillers of the fungal cell wall (Shenghui Shen et al., 2019). Glycosylated proteins anchor β (1,6)-glucan chains through glycosylphosphatidylinositol (GPI). Mannosyl proteins account for 40% of the cell wall structure. Damage to mannosyl proteins will lead to the decreased activity of proteins synthesizing the cell wall, thereby affecting the strength and integrity of the cell wall, which is of great importance to the dynamic system of fungal cell wall (Schmidt et al., 2005). The chitin–glucan complex or chitosan complex is the main component of fungal cell wall, accounting for 60% of the dry weight of the cell (Araújo et al., 2020).

The active substances in many plant essential oils are mixed inhibitors of 1, 3-β-glucan synthase (FKS-1) and chitin synthase in fungi. Diallyl disulfide downregulates the expression of the chitin synthase gene, and the treated cells have reduced chitin, damaged epidermis, and changed morphology and physiological activity (Shah et al., 2020). Previous studies found that cinnamaldehyde and eugenol could bind to the active sites of these two enzyme proteins through different amino acid residues (Ju et al., 2022). *Artemisia monosperma* Del., *Callistemon viminals* G. Don, *Citrus aurantifolia* Swingle, and *Cupressus macrocarpa* Hartw. ex Gordon essential oils also inhibited chitin production (Abdelgaleil and El-Sabrout, 2018). Some experiments have found that a sub-inhibitory concentration of cinnamaldehyde can directly or indirectly attack the target of azole, polyene, and echinocandin through the accumulation of reactive oxygen species to induce moderate downregulation of the transcription of ERG-2, ERG-3, ERG-4, and ERG-11, which is consistent with the change trend of ergosterol (Parks and Casey, 1995). The upregulation of sterol influx transporters (such as AUS-1 and TIR-3) and sterol metabolism regulators (such as SUT-1 and UPC-2) inhibits ergosterol in a dose-dependent manner and then affects cell membrane integrity (Li Q. Q. et al., 2018). Estragole and linalool were found to inhibit ergosterol in a similar way as azole antifungal agents, both of which showed an inhibitory effect on 14α-demethylase, a key enzyme in ergosterol synthesis (Khan et al., 2010).

#### 3.2.4 Damage to mitochondrial function

​ATPases are involved in most biological and physiological activities in the cell through energy coupling. When plant oils disrupt the permeability and fluidity of plasma membranes, the membrane potential of organelles is reduced; proton pumping is disrupted, and the synthesis of H-ATPase, the key enzyme in ATP production, is inhibited. Some polyphenolic compounds block the activity of ATP synthase by binding to some chemical binding cavities of the enzyme, and the functional groups of inhibitors have established interactions with key amino acid residues of the enzyme through hydrogen bonding, hydrophobic interaction, etc. (Issa et al., 2019). After the inhibition of ATPase, related dependent enzymes are also affected. The decrease of protease and phospholipase activities can be attributed to the degradation of ATPase mediated by cinnamaldehyde (Pootong et al., 2017), and the expression level of fatty acid synthases such as acetyl-CoA carboxylase and fatty acid biosynthetic enzymes (fasI, fasH, and fasF) is inhibited. In addition, glycerophospho acyltransferase (plsX, plsY, plsC), cytidine diphosphate-diacylglycerol synthase A (cdsA), phosphatidylglycerol synthase A (pgsA), cardiolipin synthase (cls), and polypeptide resistance factor (mprF) glycerophospholipid biosynthesis pathways are inhibited; thus, the degree of damage of bacterial biofilm is aggravated (Pang et al., 2021). After cinnamaldehyde treatment, the ATP permeability of biofilms was increased; the ATP level in biofilms was remarkable decreased, and the intracellular ATP was rapidly consumed to maintain pH. After eugenol and citral treatment, *Pseudomonas roqueforti* was found to have an altered mitochondrial morphology and membrane potential, which further damaged the metabolic pathways of cellular energy and finally induced apoptosis (Ju et al., 2020).

#### 3.2.5 Inhibition of filamentous temperature-sensitive proteins

The filamentous temperature-sensitive protein Z (FtsZ) is a GTPase with weak a sequence homology with tubulin, which plays an active role in guiding the binary division of bacteria. The FtsZ is assembled into a Z-ring structure at the future cell division site. The Z ring can promote cytodivision and recruit more than a dozen other division proteins into the Z ring. As the Z ring contraction leads to membrane closure, the cell divides to form two daughter cells (Osawa et al., 2008). The inhibition of the FtsZ by most plant oils is related to GTPase activity, FtsZ binding capacity, Z-ring assembly, and contraction. The majority of FtsZ inhibitory compounds in essential oils are phenylpropanoids and polyphenols. Cinnamaldehyde can bind to the T7 loop in the C-terminal region of the FtsZ monomer, which interferes with the formation of the Z ring and disrupts its morphology *in vivo*. The formation of the cell membrane is incomplete, and it is in a filamentous structure that is not completely divided (Domadia et al., 2007). By interacting with the GTPase binding cavity, curcumin enhanced the GTPase activity and interfered with the assembly and polymerization of the FtsZ, thereby shortening the steady-state duration of polymer assembly (Duggirala et al., 2014). Totarol is an bacteriostatic and fungistatic active ingredient derived from the plant *Rhamnosus pinus*. After its treatment, the cell GTPase activity and FtsZ polymerization are inhibited; the cells are filamentous, and the Z-ring assembly is misaligned (Jaiswal et al., 2007). Germarene and gemmarene D-4ol in pine needle essential oil can also bind to the hydrophobic cavity of the FtsZ (Anderson et al., 2012).

#### 3.2.6 Interference with microbial gene expression

​Nucleic acid is the material in which genetic information is stored, copied, and transmitted. The expression of genetic material controls the synthesis and metabolism of cellular proteins. Therefore, nucleic acid and gene expression are also important antimicrobial pathways. *Listeria monocytogenes* were treated with lemongrass essential oil to observe their transcriptome response, and the virulence genes *hly* and *inlj* were downregulated in a dose-dependent manner, whereas the fatty acid biosynthesis gene *accP* was upregulated (Hadjilouka et al., 2017). Fennel essential oil downregulates genes related to ochratoxin A (Nowotarska et al., 2017) biosynthesis in *A. niger*, thereby reducing OTA levels, but no changes in fungal spore morphology were observed, so fennel essential oil may only inhibit OTA production by reducing the expression of virulence-related genes (El Khoury et al., 2016). The test of citrus essential oil on *S. aureus* also showed that the expression of cytotoxic genes (*comC*, *comD*, *gtfB, gffC, and gbpB*) was remarkably downregulated, and its main active components, namely, linalool and limonene, indirectly inhibited the production of glucans required for biofilm formation (Benzaid et al., 2021). Transcriptome analysis showed that the indirect exposure of essential oil could affect the expression of *Penicillium rubens* genes, and essential oil could inhibit the activity of *P. rubens* by affecting polysaccharide, carbohydrate, fatty acid, nucleotide, and nucleoside metabolism (Kisová et al., 2020).

## 4 Plant essential oil regulates the fermentation quality of feed silage

​The quality of silage feed largely depends on the ability to preserve the nutritional components of the silage raw material. After the plant has been harvested, microbial and plant cell respiration are the main sources of nutrient loss. Therefore, silage should be carried out as soon as possible after the plant is harvested, using lactic acid bacteria to produce organic acids, lower the pH of the silage environment, inhibit the growth of harmful microorganisms, and reduce the loss of raw material nutrients. However, in the early and late stages of plant silage and after the silage feed is opened, the quality of silage feed is often affected by harmful microorganisms such as mold. The characteristic of essential oils that have strong inhibitory effects on mold at equal concentrations but are not remarkably inhibitory to lactic acid bacteria meets the needs of silage. Therefore, plant essential oils can be applied to silage feed to improve silage quality. (Bolsen et al., 1996).

### 4.1 Inhibition of growth and reproduction of adverse microorganisms in silage

The quality of silage depends on the microbial community of the silage raw material and the succession of microbial colonies during fermentation. However, adverse factors such as loose sealing measures and low sugar content in silage production may lead to changes in microbial community structure in silage affected by Clostridium, yeast, mold and other spoilage microorganisms, thereby reducing the quality of silage (Dunière et al., 2013; Xin et al., 2021). Clostridia can grow in an anaerobic environment, especially in high-moisture forage, and can compete with LAB. It has been reported that *Bacteroidetes* such as *Palidibacter propionicigenes* and *Prevotella ruminicola* have better ability to degrade small molecular substances than *Firmicutes*. It is an important factor in the loss and degradation of small molecular substances such as monosaccharides, disaccharides and non-cellulosic polysaccharides in silage (Klang et al., 2015). Spoilage microorganisms, such as mold and yeast, usually multiply in large numbers during the secondary fermentation of silage, degrading the nutritional quality of silage, affecting the palatability and producing a series of harmful substances: aflatoxins, ochratoxins, trichothecenes, fumonisins, and mycophenolic acids (Haq et al., 2021). In reducing the growth and reproduction of harmful microorganisms in early silage, adding appropriate amount of plant essential oil to inhibit the growth and reproduction of harmful microorganisms can improve the quality of silage. Several experiments have shown that cinnamon essential oil (*C. zeylanicum*), thyme essential oil (*Thymus mongolicus*), oregano essential oil (*Origanum minutiflorum*) (Foskolos et al., 2016; Çayıroğlu et al., 2020), cumin essential oil (*Cuminum cyminum*) (Turan and Önenç, 2018), lemon seed essential oil(*Citrus limon*) (Besharati and Niazifar, 2020), lemongrass essential oil (*C. citratus*) (Júnior et al., 2020), flaxseed essential oil (*Linum usitatissimum*), *Amomum villosum* Lour essential oil (Li et al., 2022), and sweet orange essential oil (*Amomum villosum Lour.*) (Hodjatpanah-Montazeri et al., 2016; Chaves et al., 2021) significantly inhibited the growth of Proteobacteria, Bacteroidetes and Actinobacteria, which provided a favorable environment for the growth of Firmicutes. The addition of cinnamon essential oil in silage reduced the relative abundance of unfavorable silage bacteria, such as Proteobacteria, Bacteroidetes and Actinobacteria, and increased the relative abundance of Firmicutes (Sheng-tan et al., 2011). After the silage was opened and fermented, the number of fungus and yeast colonies in the silage supplemented with cumin essential oil (Turan and Önenç, 2018), cinnamon essential oil (*C. zeylanicum*), oregano essential oil (*Origanum minutiflorum*) and sweet orange essential oil (*Citrus sinensis*) (Chaves et al., 2012) decreased significantly. The results showed that the addition of plant essential oil inhibited the proliferation of mold and yeast and prevented aerobic decomposition.

### 4.2 Inhibition of mycotoxin production in silage

​Mycotoxins are a group of secondary metabolites secreted by fungal organisms belonging to the genera *Aspergillus*, *Fusarium*, *Alternaria*, and *Penicillium*. The ingestion of mycotoxins by animals affects feed intake, livestock product production, neurological activity, hormone levels, and immune capacity (Ogunade et al., 2018). Although ruminal microflora of ruminants has a certain mycotoxin degradation capacity, intake of feed containing high levels of mycotoxins will also have adverse effects on animal health and livestock product production because of the saturation of ruminal detoxification capacity (Cheli et al., 2013). Studies have shown that plant essential oils can reduce the content of mycotoxins in silage, and the use of lemongrass (*C. citratus*), turmeric (*C. longa*), mint (*Mentha canadensis Linnaeus*), rosemary (*Rosmarinus officinalis*), rose grass (*Cymbopogon martini*), and other essential oil extracts can inhibit the production of a variety of mycotoxins at a certain concentration (Bryła et al., 2022). *Hedychium spicatum* L. essential oil can remarkably reduce the enol concentration (DON) and zearalenone (ZEA) content of *F. deoxynivalene* (Kalagatur et al., 2018a). Ylang-ylang essential oil (*Cananga odorata (Lam.) Hook. F. and Thomson*) at 3.9 mg/g can completely inhibit the production of DON and ZEA mycotoxins in corn containing *F. graminearum* (Kalagatur et al., 2018b). Further research has found that some plant essential oils can directly reduce mycotoxins and destroy their toxicity. Of these, *Cinnamomum cassia* is the most efficient at degrading Fumonisin B1, followed by citral (*C. limon*), *S. aromaticum*, eucalyptus (*Eucalyptus* spp.), and camphor (*Cinnamomum camphora L.*) essential oils. However, lemon, grapefruit, eucalyptus, and palm essential oils had the highest degradation efficiency of ZEA (Perczak et al., 2019). The levels of ochratoxin A (Nowotarska et al., 2017) were below the limit of detection and the levels of ZEA, DON and T-2 toxins (Trichothecenes) were significantly reduced in corn silage treated with 3 mg/kg of oregano (*O. vulgare.*) ethanol extract and 6 mg/kg of thyme (*T. vulgaris*) ethanol extract, respectively (Vaičiulienė et al., 2020). When the ethanolic extract mixture of oregano (*O. vulgare*) and thyme (*T. vulgaris*) was involved in the silage of whole corn, the levels of ZEA and DON mycotoxins, as well as T-2 toxins, in the two above mentioned plant extract mixtures were remarkably lower than those in the other experimental groups (Vaičiulienė et al., 2022).

### 4.3 Improving the feeding quality of silage

Reducing the loss of nutrients from plant raw materials is an important role of silage. During silage fermentation, plant nutrients such as protein and starch are partially degraded by plant enzymes into soluble nitrogen and soluble carbohydrates, and this reaction continues until the pH drops below 4.0. Enzymes are key factors in the degradation of nutrients such as protein and starch during silage, among which bacterial enzymes are the main cause of proteolysis (60%), followed by plant proteases (30%); fungal enzymes and fermentation products contribute about 5% of proteolysis during fermentation (Junges et al., 2017). The use of essential oils as a silage additive can indirectly reduce nutrient loss during silage. After adding 200 mg/kg of cumin (*C. cyminum*) essential oil, the colony structure of wild oat silage changes considerably, and the lactic acid bacteria multiply in large quantities to produce acid, reduce the pH value, and then inhibit the activity of protein degradation enzymes, thereby reducing the degradation of proteins (Akinci and Önenç, 2021). In addition, the results showed that plant oils directly inhibit the activity of bacterial amylases and proteases. Oregano essential oil shows strong inhibitory properties on enzymes that degrade nutrients, such as tyrosinase, α-amylase, and α-glucosidase (Sarikurkcu et al., 2015). Phenylalanine ammonia-lyase can also catalyze the deamination of L-phenylalanine, and this enzyme is an important factor in plant browning. The research results show that phenolic essential oils such as cinnamaldehyde and carvacrol can inhibit the activity of phenylalanine ammonia lyase through hydrogen bridges and ionic or hydrophobic interactions, thereby reducing the loss of feed nutrients (Foskolos et al., 2016). The silage with 50 mL/kg of cinnamon (*C. cassia*) essential oil showed the lowest ammonia nitrogen level (Hodjatpanah-Montazeri et al., 2016). Cumin (*C. cyminum*) essential oil, lemongrass (*Cymbopogon citratus*) essential oil with a low concentration level, lemon seed (*C. limon*) essential oil, oregano (*O. vulgare*) essential oil, and flaxseed (*L. usitatissimum*) essential oil inhibited the production of ammonia nitrogen, propionic acid, and butyric acid during silage (Turan and Önenç, 2018; Çayıroğlu et al., 2020; Besharati and Niazifar, 2020; Besharati et al., 2020; Júnior et al., 2020). Plant essential oil extracts such as cinnamon (*Cinnamomum cassia L.*) essential oil, lemongrass (*C. citratus*) essential oil, flaxseed (*L. usitatissimum*) essential oil, clove (*E. caryophyllata Thunb.*) essential oil, thyme (*T. mongolicus*) essential oil, *A. villosum* Lour essential oil, lemon (*C. limon*) seed essential oil, and flaxseed (*L. usitatissimum*) essential oil with appropriate content as silage additives improved the evaluation level of silage and reduced the loss of crude protein, crude fat, and soluble sugar (Hodjatpanah-Montazeri et al., 2016; Turan and Önenç, 2018; Çayıroğlu et al., 2020; Besharati and Niazifar, 2020; Besharati et al., 2020; Júnior et al., 2020; Chaves et al., 2021; Li et al., 2022).

### 4.4 Improving the aerobic stability of silage

Given its wide application, silage can easily produce secondary fermentation in an aerobic environment after opening the bag (open the cellar), and aerobic microorganisms multiply in large numbers, consume nutrients in the feed, and produce a lot of heat. Therefore, the aerobic stability of silage after prolonged opening of the bag (open the cellar) is important (Wilkinson and Davies, 2013). Plant essential oil remarkably extended the aerobic stability of silage because of its excellent bacteriostatic and fungistatic activity. Studies showed that 0.13 mL/cm^2^ of oregano(*O. vulgare*) essential oil was sprayed in beet pulp (Çayıroğlu et al., 2020). Alfalfa was supplemented with 60 mg/kg of lemon seed(*C. limon*) essential oil or 60 mg/kg of flaxseed(*L. usitatissimum*), cinnamon(*C. cassia*) seed, and lemon seed(*C. limon*) mixed essential oil (Besharati and Niazifar, 2020). The addition of 120 mg/kg of cinnamon essential oil(*C. cassia*), thyme essential oil(*T. vulgaris*), oregano essential oil(*O. vulgare*), or cumin essential oil(*C. cyminum*) to corn silage can improve the aerobic stability of silage (Hodjatpanah-Montazeri et al., 2016). Cinnamon(*C. cassia*) essential oil, sweet orange(*Citrus sinensis*) essential oil, oregano(*O. vulgare*) essential oil, and thyme(*T. vulgaris*) essential oil at a level of 120 mg/kg can maintain aerobic stability for 2 weeks (Chaves et al., 2021), whereas the blank control group without added essential oil can only maintain aerobic stability for 72 h. Peppermint(*Mentra piperita*) essential oil has also been proven to have an inhibitory effect against *F. oxysporum* and *Costomyces in vitro*, and the aerobic stability time of silage was prolonged by 50 h after adding peppermint essential oil to silage (Moghaddam et al., 2013).

## 5 Summary

Plant essential oils have strong inhibitory activity against a variety of adverse microorganisms in silage. Plant essential oil achieves its bacteriostatic and fungistatic ability by inhibiting biofilm formation, changing cell membrane permeability, and interfering with cell division and ATPase activity. The difference in bacteriostatic and fungistatic activity of plant essential oil against some fungi and lactic acid bacteria lays a theoretical foundation for its application as feed additives in silage. The use of appropriate levels of plant essential oils in silage production can control changes in colony structure by inhibiting the growth of some adverse microorganisms such as Clostridium, Fusarium and yeast in silage, and indirectly promote lactic acid bacteria to become dominant microorganisms. Reduce nutrient loss in silage, improve fermentation quality, and improve the aerobic stability of silage after secondary fermentation in the later stage.

## Author contributions

LC: Writing – original draft, Writing – review & editing. XL: Writing – original draft, Writing – review & editing. YW: Writing – review & editing. ZG: Writing – review & editing. GW: Writing – review & editing. YZ: Writing – review & editing.
